# Evaluating Biodiversity Metric Response to Forecasted Land Use Change in the Northern Rio Grande Basin

**DOI:** 10.3390/environments5080091

**Published:** 2018

**Authors:** Elizabeth A. Samson, Kenneth G. Boykin, William G. Kepner, Mark C. Andersen, Alexander Fernald

**Affiliations:** 1 Center for Applied Spatial Ecology, New Mexico Cooperative Fish and Wildlife Research Unit, Department of Fish, Wildlife, and Conservation Ecology, New Mexico State University, Las Cruces, NM 88003, USA; 2 U.S. Environmental Protection Agency, Office of Research and Development, Las Vegas, NV 89119, USA; 3 Department of Fish, Wildlife, and Conservation Ecology, New Mexico State University, Las Cruces, NM 88003, USA; 4 Department of Animal and Range Science, New Mexico State University, Las Cruces, NM 88003, USA

**Keywords:** biodiversity, ecosystem services, land use change, wildlife species, urban growth, deductive habitat models, wildlife habitat, biodiversity metrics, land use scenarios, Rio Grande River

## Abstract

The effects of future land use change on arid and semi-arid watersheds in the American Southwest have important management implications. Seamless, national-scale land-use-change scenarios for developed land were acquired from the US Environmental Protection Agency Integrated Climate and Land Use Scenarios (lCLUS) project and extracted to fit the Northern Rio Grande River Basin, New Mexico relative to projections of housing density for the period from 2000 through 2100. Habitat models developed from the Southwest Regional Gap Analysis Project were invoked to examine changes in wildlife habitat and biodiversity metrics using five ICLUS scenarios. The scenarios represent a US Census base-case and four modifications that were consistent with the different assumptions underlying the A1, A2, B1, and B2 Intergovernmental Panel on Climate Change global greenhouse gas emission storylines. Habitat models for terrestrial vertebrate species were used to derive metrics reflecting ecosystem services or biodiversity aspects valued by humans that could be quantified and mapped. Example metrics included total terrestrial vertebrate species richness, bird species richness, threatened and endangered species, and harvestable species (e.g., waterfowl, big game). Overall, the defined scenarios indicated that the housing density and extent of developed lands will increase throughout the century with a resultant decrease in area for all species richness categories. The A2 Scenario, in general, showed greatest effect on area by species richness category. The integration of the land use scenarios with biodiversity metrics derived from deductive habitat models may prove to be an important tool for decision makers involved in impact assessments and adaptive planning processes.

## 1. Introduction

With global increases in human population growth, there is a need for research regarding the relationship between population induced land use change and biodiversity [1,2]. Human induced changes to biodiversity have occurred more rapidly in the past 50 years than at any other time in history, and the forces driving such changes are steady, showing no evidence of decline over time [3]. In the past, conservation efforts have largely focused on conserving individual species and habitats. More recently, conservation ecologists have focused on ecosystem services or the goods and services from ecological systems that benefit people [3]. Consequently, research has focused on the role of biodiversity in ecological service functioning and maintenance [4,5]. Humans directly depend on ecosystem services; however, they are continually altering the landscape, leading to biodiversity degradation and loss of ecosystem integrity [6]. Ecosystem services are diminishing due to increased ecosystem alterations in response to higher demands and increasing populations [7,8]. The ecosystem services concept suggests that conservation efforts should focus on ecosystems and landscapes instead of individual species to preserve biodiversity and ensure the availability of these goods and services [9–11] into the future.

Changes in ecosystems can alter the provision of ecosystem services [3]. The link between ecosystem functioning and biodiversity is not well understood, but studies have demonstrated that high levels of biodiversity are linked to ecosystem productivity and thus, the production of ecosystem services [11–13]. Biodiversity can be affected both directly and indirectly by human population growth, land use change, and climate change, among other factors [1,14,15]. Direct effects include the loss or fragmentation of habitat. Indirect effects include the change of processes or functions (e.g., snowpack and surface flow) that affect the maintenance of habitat and species. The link between wildlife and ecosystem services has been a topic of several studies [5,11]. For example, endangered species habitats have decreased directly due to urban development [16]. Previous studies have highlighted a need for continuing research on the effect of land use change on ecosystem services, biodiversity, and wildlife habitats [1,5,11,17–19]. Concurrently, there is a need to understand the value of biodiversity as an ecosystem function and to find the balance between multiple ecosystem services [11,20–22].

In the Southwestern United States, riparian areas provide critical ecosystem functions and are associated with high levels of biodiversity [18,23–29]. Riparian systems are under pressure from shifting management practices, population growth, urbanization, and climate change [30,31]. Functions and services from these spatially limited systems are also affected by stressors within the adjacent watersheds. The traditional acequia systems in Northern New Mexico provide a similar focal point for biodiversity, ecosystem function, and ecosystem services [32–34]. Acequias are a network of traditional gravity fed, unlined irrigation ditches that mirror natural hydrological and ecological functions and aid in the maintenance of ecosystem services [31,34]. Similar to natural riparian systems, these artificial historic systems provide hydrological processes that create distinctive vegetation communities both in composition and structure [33]. This increase in riparian vegetation provides wildlife with an additional habitat within this limited system. However, the effect of urban growth through alternative future scenarios may highlight the need for additional acequia maintenance and management.

Studies of alternative futures aim to evaluate the effects that different policies, management, and scenarios have on biodiversity and ecosystem services [30]. The United States Environmental Protection Agency (EPA) created the Integrated Climate and Land-Use Scenarios (ICLUS) database to study the future impacts of population growth and urban development [35]. Our objective was to determine the effects of urban development and growth on biodiversity and to select ecosystem services within the Northern Rio Grande Basin based on five alternative future scenarios. We identified species-rich areas within the Basin, and then investigated the effect of future land-use on species richness. We compared future land-use scenarios in the Northern Rio Grande river basin using the ICLUS dataset to measure future urban grow-out effects on four biodiversity metrics [5] derived from the Southwest Regional Gap Analysis Project (SWReGAP) deductive habitat models [36]. Our effort quantifies the extent and magnitude of the changes based on alternative future scenarios.

## 2. Materials and Methods

### 2.1. Study Area

**Figure 1.**
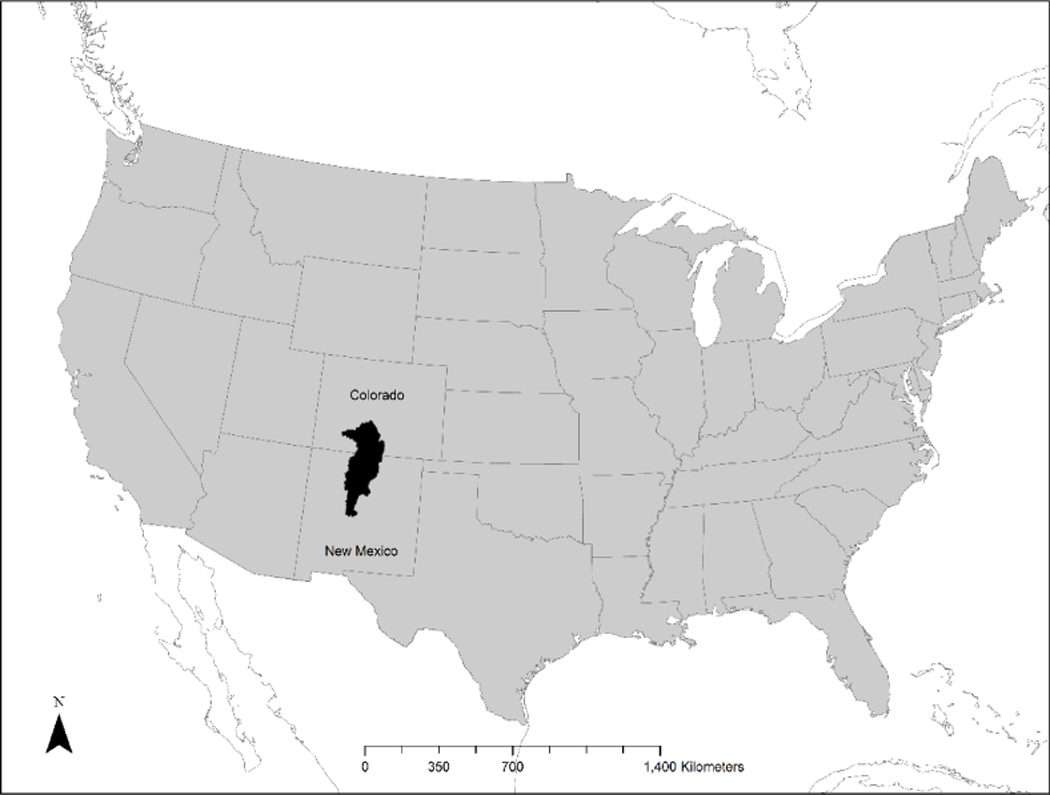
Map of the study area: the Northern Rio Grande River Basin beginning with the headwaters in Colorado, flowing south into New Mexico.

The study area was the Northern Rio Grande River Basin (Figure 1) which includes the cities of Albuquerque (population 556,092), Rio Rancho (population 91,807), and Santa Fe (population 83,008: [37]). This watershed is projected to be in the top 40 highest growing regions in the United States by 2030 [38]. The study area stretches from the Rio Grande headwaters in Colorado, south to the confluence with the Rio Puerco downstream of Albuquerque. The study area encompasses an area of approximately 50,378 km^2^. Elevations range from 1400 to 4350 m above sea level with precipitation increasing with elevation [22]. Major vegetation types in the area, as mapped by SWReGAP, include Southern Rocky Mountain Pinyon-Juniper Woodland, Rocky Mountain Ponderosa Pine Woodland, Inter-Mountain Basins Semi-Desert Shrub Steppe, and Inter-Mountain Basins Semi-Desert Grassland [23].

### 2.2. Datasets

The EPA-ICLUS (Version 1.3.1) dataset was used to assess habitat change and effects on biodiversity metrics. These seamless, national-scale land-use change scenarios for developed land were acquired from EPA’s Office of Research and Development [35]. The data were extracted from the national coverage for the Northern Rio Grande Basin. This dataset allowed for analysis of projections of housing density for the period from 2000 through 2100 for the five ICLUS scenarios, including a US Census baseline and four modifications consistent with the different assumptions underlying the A1, A2, B1, and B2 Intergovernmental Panel on Climate Change (IPCC) global greenhouse gas emission storylines (Table 1; [35]). The five ICLUS datasets were reclassified to identify urban (1) or nonurban areas (0). The ICLUS national-scale housing-density (HD) scenarios are consistent with the Intergovernmental Panel on Climate Change (IPCC 2001) Special Report on Emissions Scenarios (SRES; [39]) greenhouse gas emissions storylines and they are available in 10-year increments until 2100.

Within the SRES, the A2 scenario is characterized by a high fertility rate (average number of children that would be born to a woman over her lifetime) and low net international migration; it represents the highest US population scenario gain (690 million people by 2100), whereas the Base Case (BC) and B2 scenario are characterized as middle scenarios with medium fertility rates and medium to low levels of international migration. The differences between BC and B2, as well as A1 and B1, reflect how housing is allocated, i.e., sprawl vs. compact growth patterns. The primary difference between these scenarios occurs at the domestic migration level with an assumption of high domestic migration under A1 and low domestic migration under B1. The effect of different migration assumptions becomes evident in the spatial model when the population is allocated into housing units across the landscape. The national US Baseline forecast for 2100 is 450 million people and B1 is lower at 380 million people. The A2 scenario results in the largest changes in urban and suburban housing density classes and greater conversion of natural land-cover classes into new population centers or urban sprawl. The largest shift from suburban densities to urban are typically predicted to occur between 2050 and 2100 for the A-family.

The United States Geological Survey developed regional level datasets focusing on biodiversity conservation through the Gap Analysis Program (GAP [40]). GAP mapped land cover and other environmental factors such as landforms, and elevation to predict suitable habitats for all terrestrial vertebrates. SWReGAP created 817 species-level habitat models for Arizona, Colorado, Nevada, New Mexico, and Utah [36].

### 2.3. Analysis

We extracted ICLUS data for the regional study area and reclassified it into urban and nonurban areas. The ICLUS dataset identified 13 land cover classifications that were aggregated into four classifications. The classification was based on acres per housing unit as urban (<0.25 acres/housing unit), suburban (0.25–2 acres/housing unit), and exurban (2–40 acres/housing unit). Areas with more than 40 acres/housing unit were considered rural or natural areas [24]. For further analysis, we combined the urban, suburban, and exurban types into an urban type and rural and natural areas into a nonurban type.

We derived four biodiversity metrics from 817 terrestrial vertebrate habitat models developed by SWReGAP [5,36,42]. The four metrics were (1) all terrestrial vertebrate species richness, (2) bird species richness, (3) number of harvestable species, and (4) number of threatened and endangered species. The four selected biodiversity metrics represent ecological services or aspects of biodiversity [5]. All terrestrial vertebrate species richness is a summation of all the terrestrial species that could occur in each pixel of the watershed out of 817 species modeled throughout the southwest United States [36]. Vertebrate species richness represents a surrogate for all biodiversity. Bird species richness is a summation of all bird species that could occur in each pixel. Bird species richness represents the value that the birding community has placed on bird fauna for cultural and aesthetic reasons [43]. Richness may represent the economic value in terms of birding associated tourism, insect pest control, or seed dispersal [43]. Harvestable species are listed by state wildlife agencies to be of consumptive use through hunting, trapping, or other harvest. Harvestable species not only provide food for hunters but also can provide recreational value for hunters and non-hunters. Hunting can also stimulate local economies through guide fees, outfitting, tourism, and other expenditures. Species were identified from reviewing state wildlife agency game regulations. The Federal government under the Endangered Species Act [44] lists threatened and endangered species (T&E). Threatened and endangered species are valued by society and given legal priority in management. Species were identified from US Fish and Wildlife Service list. Metrics were the summation of the species models identified for each metric. Summations were completed in geographic information systems (GIS) software (ESRI ArcMap 10.0).

We calculated and plotted the area in square kilometers for each individual richness value to identify the amount of species richness by area. We then calculated and plotted the area for each richness value as the sum of the area of all smaller richness values for area-species richness. Area-species richness curves were created to classify each biodiversity metric into five species richness categories including low species richness (1), moderate–low species richness (2), moderate species richness (3), moderate–high species richness (4), and high species richness (5). Low species richness was identified as the first inflection point of the curve, moderate–low species richness was the point between the first and second inflection points. Moderate species richness was identified as relatively constant increases in species area. The moderate–high species richness was defined as the area between the third and four inflection points or rounding of the curve. The high species richness category was identified as the portion of the curve that plateaued.

Beginning with the baseline condition (year 2000), the areas of each species richness category for each biodiversity metric were quantified for nonurban and urban land cover types for each of the five ICLUS scenarios using GIS software (ESRI ArcMap 10.0). The change in area (km^2^) and relative percentages of land classified as natural or urban were calculated for the four biodiversity metrics for the year 2000 and 2100 under the five ICLUS future scenarios for the study area. The underlying species models and resultant biodiversity metrics were based on 2001 imagery and were not available for prediction for 2100. The four biodiversity metrics did not change over time.

## 3. Results

### 3.1. Natural Areas

The amount of natural area converted to the three categories of urban areas (exurban, suburban, and urban) was calculated for each of the five ICLUS scenarios (Table 2). As expected, the A2 scenario had the greatest change with a loss of 19,537 km^2^ (3.49%) of natural areas. The A1 scenario had less than half the loss of A2 and the other three scenarios had losses of less than 0.28% (B1, B2, and BC).

### 3.2. Species Richness

**Figure 2.**
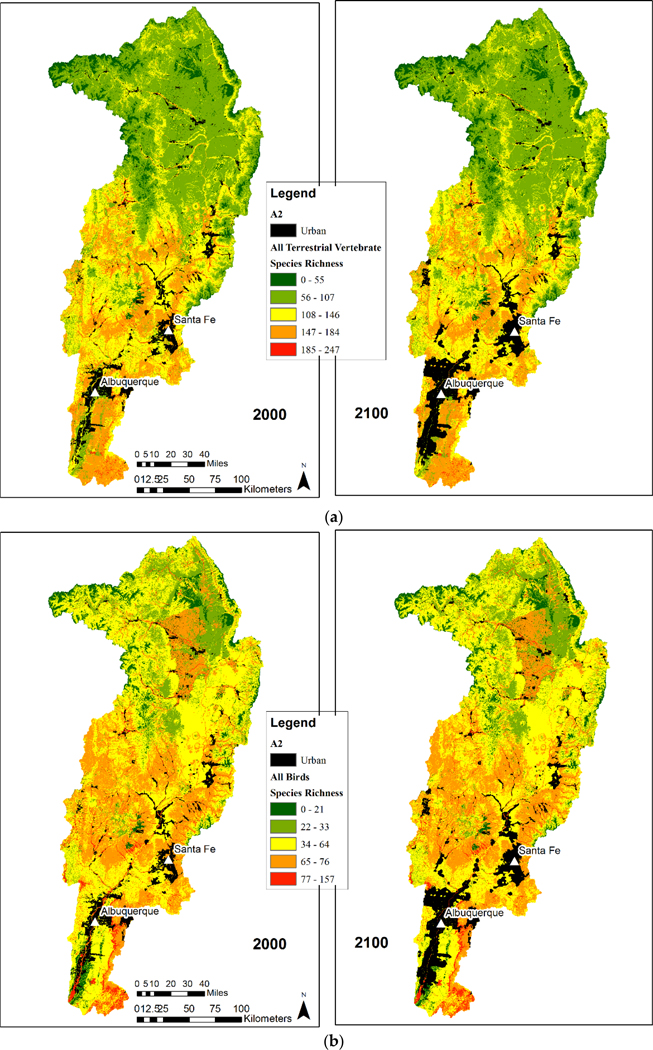
Urban area grow-out under scenario A2 for the years 2000 and 2100 for (**a**) all terrestrial vertebrate species richness and (**b**) all bird species richness within the upper Rio Grande Basin in New Mexico.

**Figure 3.**
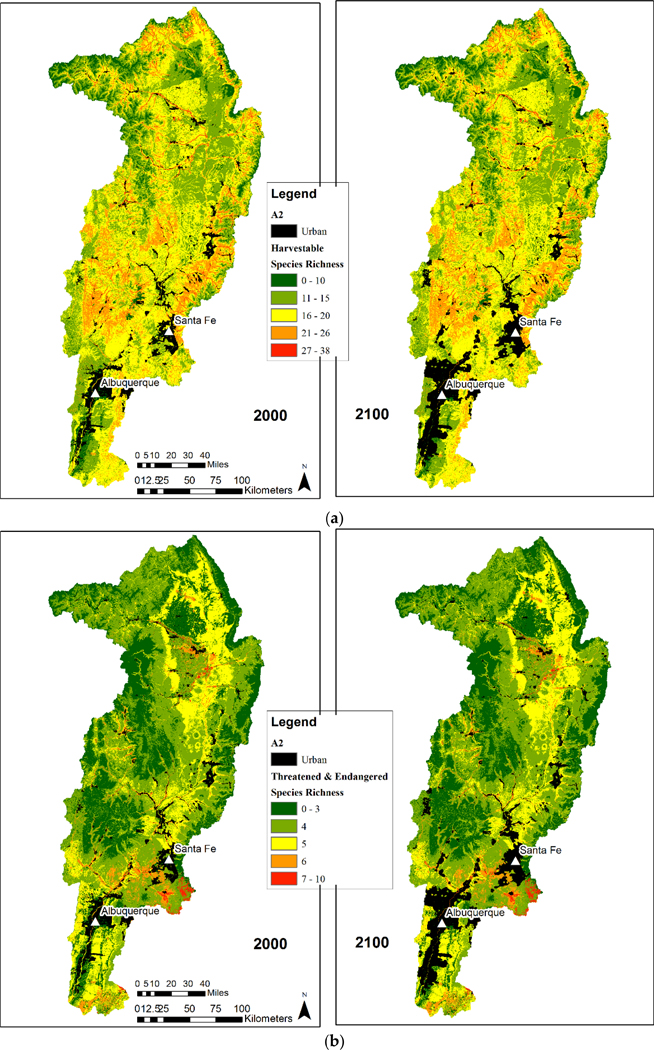
Urban area grow-out for scenario A2 for the years 2000 and 2100 for (**a**) number of harvestable species, and (**b**) number of federally listed threatened and endangered terrestrial vertebrate species within the upper Rio Grande Basin in New Mexico.

The spatial distributions of the four metrics provided insight into the effect of urbanization on species richness (Figures 2 and 3). Species richness was shown to be highest for all terrestrial vertebrate species and all bird species metrics at the lower (Southern) end of the watershed. This area is also in closest proximity to urban areas including Albuquerque and Santa Fe, New Mexico. As the urban areas expand, these species rich areas are at most risk and are projected to incur losses.

**Figure 4.**
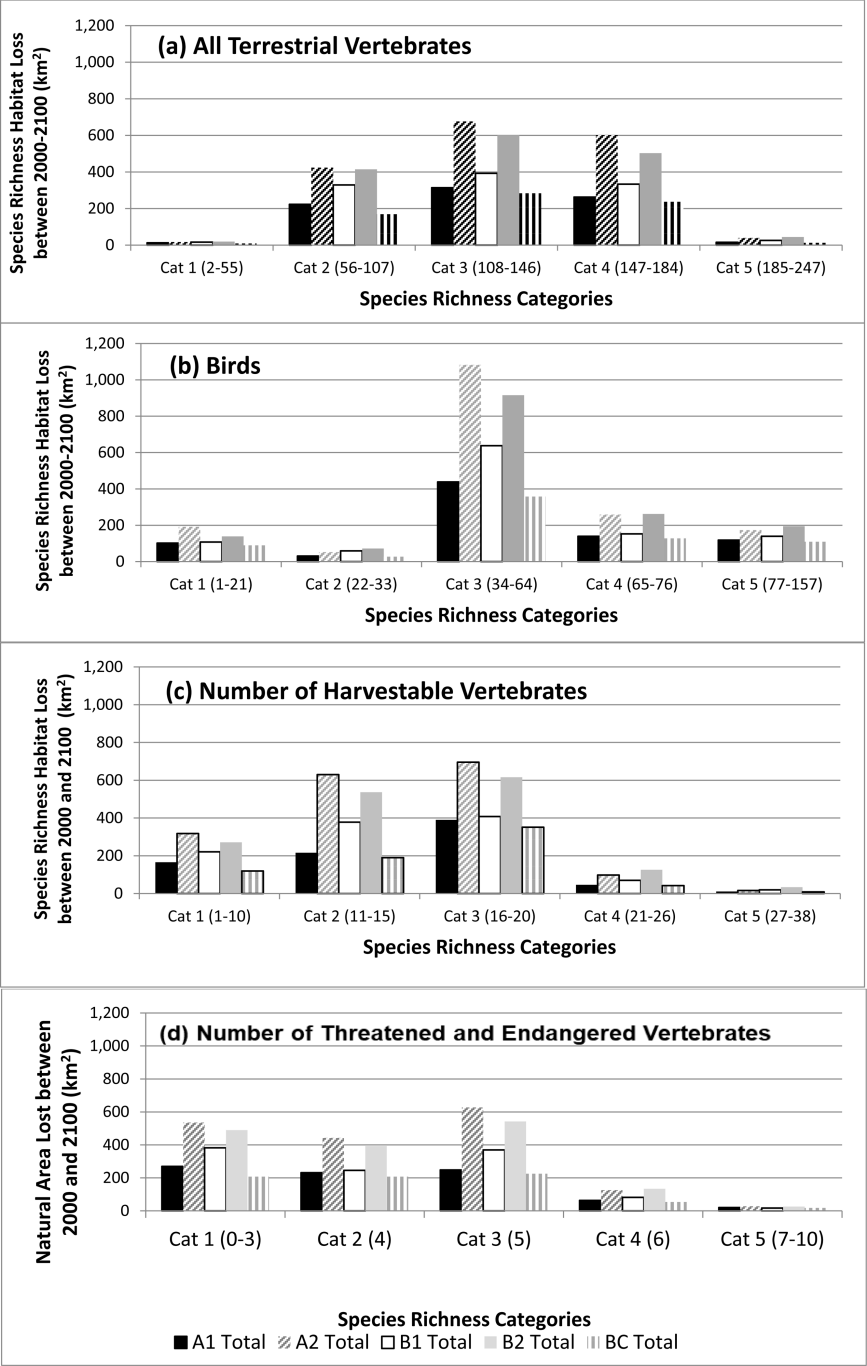
Square kilometers of loss of (**a**) all terrestrial vertebrate species, (**b**) birds, (**c**) number of harvestable species, and (**d**) number of threatened and endangered species under five Integrated Climate and Land-Use Scenarios [24] by richness category from 2000 to 2100. The number of species in each richness category are provided in parentheses.

**Figure 5.**
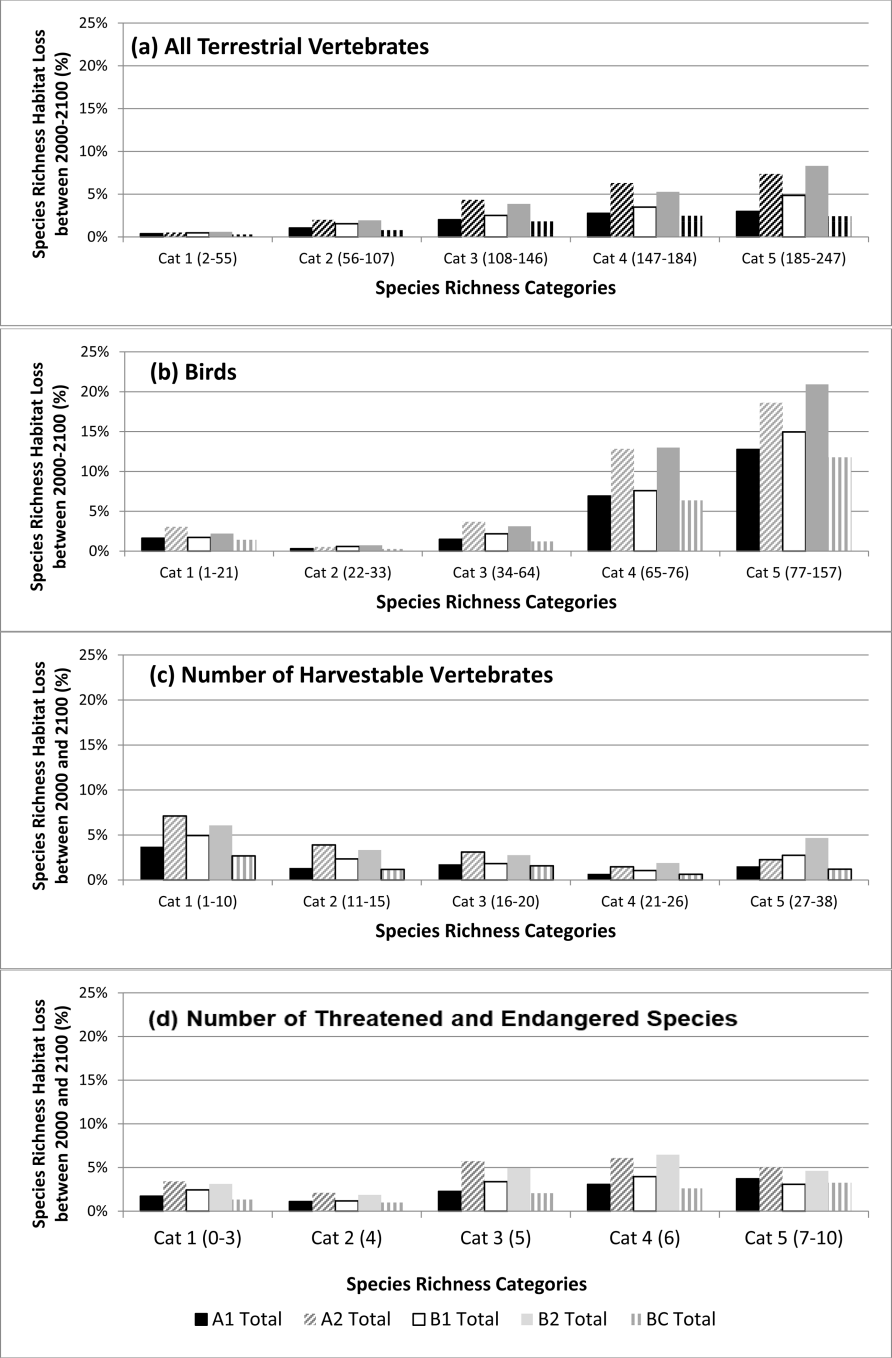
Relative percent loss of (**a**) all terrestrial vertebrate species, (**b**) birds, (**c**) number of harvestable species, and (**d**) number of threatened and endangered species under five Integrated Climate and Land-Use Scenarios [24] by richness category from 2000 to 2100. The number of species in each richness category are provided in parenthesis.

**Figure 6.**
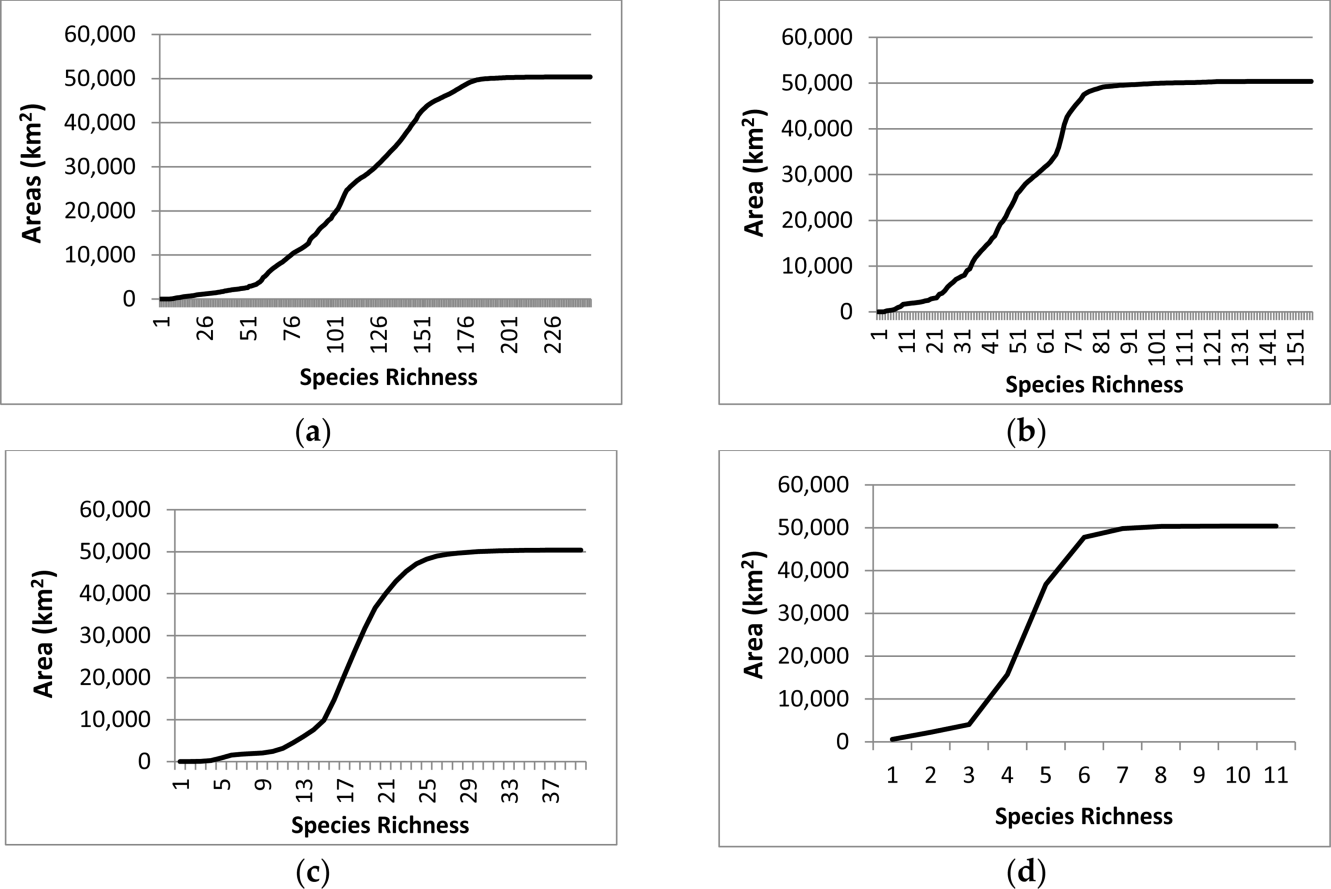
Current area-species richness curves for (**a**) all terrestrial vertebrate species richness, (**b**) bird species richness, (**c**) number of harvestable species, and (**d**) number of threatened and endangered species within the Northern Rio Grande Basin.

**Figure 7.**
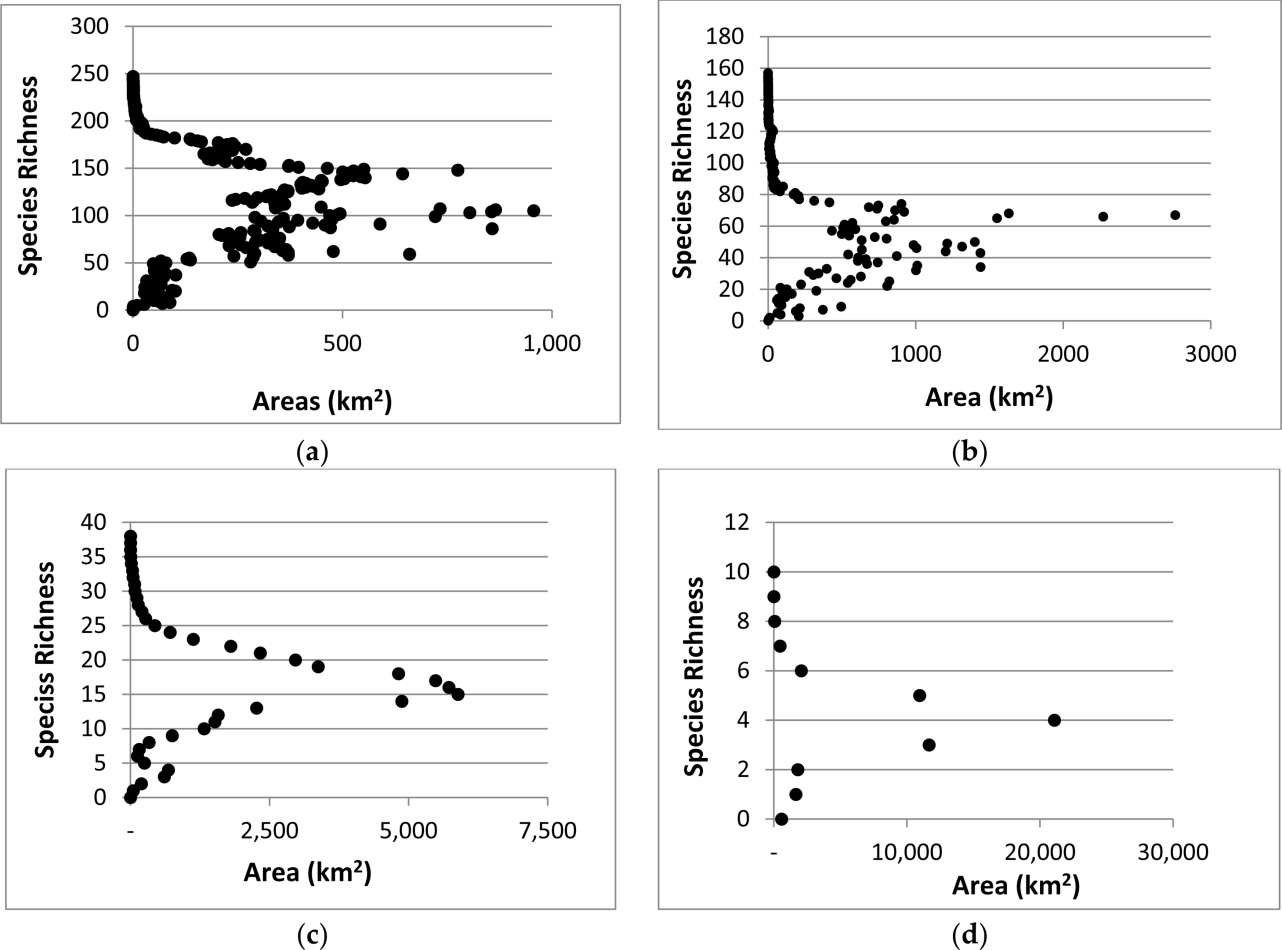
Current species richness by area for (**a**) vertebrate species richness, (**b**) bird species richness, (**c**) number of harvestable species, and (**d**) number of threatened and endangered species within the Northern Rio Grande Basin.

Total species richness was highest in ponderosa pine forests, pinyon-juniper woodlands, semi-desert grasslands, and riparian habitats and lower in the spruce-fir forests and agricultural areas of the San Luis Valley of Colorado. For each richness category and ICLUS scenario, we calculated the amount and relative percentage of natural area (habitat) converted to urban area (Figures 4 and 5). Most habitat was lost for the all terrestrial vertebrate species metric in the moderate categories (2–4). This ranged from a loss of around 200 km^2^ to over 600 km^2^, with the least and most species rich categories losing less than 50 km^2^ (Figure 6), though in terms of relative percentages, the higher richness categories (3–5) lost a greater percentage of habitat, ranging from 2 to 8% (Figure 7). This reflects urbanization with scenarios A2 and B2 consistently predicting the larger loss.

The areas with the highest and lowest total species richness occupied a small proportion of the study area and areas with moderate species richness occupied the majority of the area. This highlights a general pattern of area by species richness curves (Figure 6) with (1) species poor areas found in small areas, (2) followed by a corresponding increase in richness and area; and (3) then a plateauing curve with a large increase in species richness and a small increase in area. This was also observed when species richness was plotted by the amount of area for each richness value (Figure 6). In the study area, riparian habitats were often associated with high species richness.

Birds are distributed across the entire watershed more than terrestrial vertebrates as a whole (Figure 2). Bird-rich areas are found throughout the watershed including spruce-fir forests, ponderosa pine forests, pinyon-juniper woodlands, agriculture, semi-desert grasslands, and riparian areas. For birds, the majority of habitat loss was in category 3 for all scenarios with losses from 400 to over 1000 km^2^. All other category losses were around or under 200 km^2^. Relative percentages were higher in categories 4 (6–13%) and 5 (10–21%) (Figures 4 and 5). These richness categories (4 and 5) had losses of greater than 5% for two scenarios in category 4 (A2 and B2) and losses of more than 10% in category 5 for all scenarios (Figure 5). Similar to the total species richness, areas with the highest and lowest amounts of species richness occupied small proportions of the study area and areas with moderate species richness occupied the majority of the area. Areas with more than 80 species occupied less than 5 km^2^ (Figures 6 and 7).

Harvestable species are distributed throughout the watershed and though urbanization is expected to occur where more harvestable species occur, this was a small proportion of the area (Figure 3). We calculated the amount and relative percentage of natural area (habitat) converted to urban (Figures 4 and 5). Harvestable species habitat loss was in the low to moderate categories 1–3 (Figure 4). The range of lost habitat varied with category 1 ranging from 100 to 300 km^2^, category 2 ranging from 200 to 600 km^2^, and category 3 ranging from 375 to 650 km^2^. Three scenarios (A2, B1, B2) in the lowest richness category had losses at or over 5% with urban conversion increased to almost seven percent for scenario A2 (Figure 5). Ranges for all categories varied from 1% to 7%. Similar to the total species richness and bird richness, areas with the highest and lowest species richness occupied a small proportion of the study area and areas with moderate species richness occupied the majority of the area. Areas with 13–21 species were the only levels with habitats over 2500 km^2^ (Figures 6 and 7).

Federally listed threatened and endangered terrestrial vertebrates have several species-rich areas with one area between Santa Fe and Albuquerque (Figure 3). Calculations of the amount and relative percentage of natural area (habitat) converted to urban (Figures 4 and 5) highlights the effect of urbanization. Species in the threatened and endangered categories 1–3 (low and moderate richness) were projected to lose the most habitat in scenarios A2 and A1 (Figure 4). These categories will lose between 200 and 600 km^2^ of habitat. On a percentage basis, the three most species rich categories (3–5) were projected to lose less than 10% of their habitat (Figure 5) in scenario A2 All richness categories within these two scenarios (A2 and A1) lost under 6%. Similar to other richness metrics, the highest and lowest species richness categories were projected to occupy a small proportion of the study area and areas with moderate species richness were projected to occupy the majority of the area. Areas with 3, 4, and 5 species were the only levels with habitats over 1000 km^2^ (Figures 6 and 7).

## 4. Discussion

Analysis of land use projections identified several regionally important aspects of biodiversity for the Rio Grande Basin. First, all four scenarios identified losses in natural areas and species richness, though the magnitude of that loss was variable. Second, riparian areas were shown to be the most species rich areas within the Northern Rio Grande Basin. This supports previous work in riparian areas in the US Southwest [18,23,24,45–47].

Each land use scenario identified losses in natural areas. The Southwest is one of the fastest growing regions in the United States. The population of New Mexico is projected to grow 15.4% between 2000 and 2030 with medium emigration occurring which is consistent with scenario BC [38]. The Rio Grande has large urban areas (e.g., Albuquerque and Santa Fe) adjacent to the river or along tributaries. Much of the expected growth or disturbance will be associated with these areas. However, outlying communities will also expand. This projected increase in population will continue to increase pressure placed on natural lands, resources, and wildlife.

All four biodiversity metrics (all vertebrate species, bird, harvestable species, and threatened and endangered species) declined across each species richness category. Scenario A2 had the highest relative percent of area loss across richness categories. Scenario A2 represents a future where the population is growing more rapidly than other scenarios due to high fertility rates and slow economic growth [24]. The most species rich categories experienced larger decreases in the all vertebrate species and bird metrics with the larger decline in the bird species richness. Threatened and endangered species were projected to lose the most relative habitat particularly in Scenarios A1 and A2 where the analysis identified losses of at least 10% across all richness categories.

Each metric represents different ecosystem services or aspects of biodiversity [5]. Vertebrate species richness, as a surrogate for total biodiversity, declined for all scenarios and mirrored projections for global biodiversity loss [14]. Placing economic value on this loss is difficult; however, continued urban growth can lead to habitat loss which is a key factor when species are reviewed to be federally listed as T&E. Bird species richness declines were larger across all scenarios. Declines in bird species richness can result in declines in economic value from birding associated tourism, insect pest control, or seed dispersal [43]. Wildlife watching, which is often associated mostly with birds has been estimated to provide $56 billion dollars in related expenditures and $166 billion dollars in related economic benefit [48,49]. Harvestable species declines can affect the recreational value for hunters and non-hunters. Declines within harvestable species can also reduce the economic activity associated with hunting on local economies through direct hunting related expenditures ($33 billion) and additional economic benefit ($78 billion) through guide fees, outfitting, tourism, and other expenditures [48,49]. Declines in T&E habitats put these species under greater threat causing additional governmental funds to be invested in management. Additionally, other species such as those identified within the vertebrate species and bird species richness may warrant becoming listed.

The richest area for all species occurs within riparian habitats near the Rio Grande and tributaries where urban development is occurring most rapidly [45]. The Northern portion of the Rio Grande, primarily in the mountainous regions, is under federal management, unlike much of the riparian and lowland areas [40]. While riparian habitats may be under stress from urbanization and climate change, the mountainous forests are under stress from climate change.

Riparian habitats are transition zones between upland habitats and aquatic habitats and are driven by hydrological processes including surface flooding and groundwater flow [31]. The response to these processes is a distinct vegetation community, both in composition and structure, from the surrounding uplands [23]. Changes in these hydrological processes may affect riparian vegetation that occurs in these spatially limited (2% of the entire Southwest US) narrow bands along waterways [25]. As our analysis and others have shown, riparian areas support numerous terrestrial wildlife species including a high diversity of avian species [23,26,50,51]. Riparian vegetation provides water, forage, corridors, and refugia for terrestrial wildlife and aquatic species [27]. Of the vertebrate species known to occur in New Mexico, 479 of 867 species (55%) rely, at least in part, on riparian, wetland, or aquatic habitats for their survival [45]. Approximately 80% of all sensitive and specially classified vertebrate species in New Mexico use riparian areas or aquatic habitats during their life cycles [45].

The forests in Southwestern US have already been affected by climate change through increases in severe wildfire, insect outbreaks, and earlier snowmelt and peak runoff [52–54]. These factors influence the disturbance patterns and habitat of the wildlife species within these species rich areas. Increases in temperature changes can push species such as the American pika (*Ochotona princeps*) past critical thermal limits [55].

Federally threatened and endangered species are projected to be at the greatest threat in these scenarios. Many of these species have been listed because of threats to habitat. These species, such as the Southwestern willow flycatcher (*Empidonax traillii extimus*) and Mexican spotted owl (*Strix occidentalis lucida*), often occur in small numbers in limited or modified habitats. The Southwestern willow flycatcher occurs in riparian areas, and the Mexican spotted owl occurs in montane forest habitats. The habitat of these and other species continue to be stressed by land management practices, urbanization, or climate change.

Watersheds across the Southwestern United States are threatened by land use practices, population growth, and urbanization [30,31]. Our analysis supports research on riparian areas as well as community resilience and the sustainability of traditional acequia systems in Northern New Mexico [32–34]. Biodiversity, species richness, and related ecosystem services are supported by the continued use of acequias and the extension and maintenance of riparian habitats along these water systems. Acequia communities respond to changing dynamics, identification of potential tipping points for the sustainability of these systems, and the relationship between acequia systems, riparian vegetation, wildlife, and biodiversity [32–34].

This analysis was based on predicted habitat and urban growth scenarios. We did not include environmental factors associated with urban growth (e.g., water contaminants, air contaminants, and invasive species) that can accompany the urbanization process. Other direct and indirect impacts of urban grow-out can also increase habitat loss or loss of use. The metrics were derived from modeled habitats based on a land cover dataset circa 2000 [36,56,57]. This dataset does not account for vegetation changes between 2000 and 2100; thus, future scenarios through time were compared to the same baseline habitat data. This was a first level effort to understand the effect of land use over time on biodiversity metrics and ecosystem services. The complexity of modeling changes in vegetation and thus species over time based on land use and climate were well beyond the original scope of this work. Recent efforts have looked at land use and climate in Eastern US forests [58,59]. Vegetation changes due to climate change will likely exacerbate results. Environmental stochasticity, such as fire, flooding, or drought, could also be a factor.

## 5. Conclusions

Alternative future studies such as this are useful tools in evaluating the effects of different policies, management regimes, and scenarios on biodiversity [30]. Few projects have focused on similar questions at these scales [19,30]. Additional studies are needed to look at these broad scales across varying geographies, at finer scales, and under different future scenarios [1,18]. This research provides an example of a large-scale investigation of future urban grow-out and its impact on a watershed in the Southwestern United States. The analysis can inform managers and policy makers on potential patterns of growth and the resultant impact on biodiversity and ecosystem services. The analysis identified areas of conservation need and can assist in policy and management that proactively conserves biodiversity instead of retroactively protecting what remains. The richest areas within this study were the riparian habitats adjacent to the rivers and tributaries within the watershed. These same areas are associated with acequia systems. Focused conservation along the river, acequias, and riparian vegetation in areas of high biodiversity should help mitigate those direct impacts of urbanization.

Urbanization and climate change will continue to affect habitats and species. Planning for continued development must also take biodiversity and the resulting loss to ecosystems services into account. Our study identified not only the areas at risk based on forecasted land use scenarios but quantified the impact of varying alternative futures on all vertebrate species, bird, harvestable species, and threatened and endangered species metrics. Areas near current urban areas are at highest risk as development is predicted to expand from these sources. Land management that maintains or increases habitat such as acequia management will help to alleviate losses in other areas. The ecosystem services paradigm and metrics like these provide additional tools to inform decision makers during the planning process.

## Figures and Tables

**Table 1.** Description of the five Integrated Climate and Land-Use Scenarios [41].

Scenario	Description
Base Case (BC)	Represents a medium fertility rate, medium domestic migration, and medium international migration (US Census midline scenario).
A1	Represents fast economic growth, low population growth, and high global integration. Fertility is low with high domestic and international migration.
B1	Represents a globally integrated world but with more emphasis on environmentally sustainable economic development. Fertility and domestic migration are low, while international migration is high.
A2	Represents continued economic development with more regional focus and slower economic convergence between regions. Fertility and domestic migration are high and international migration is medium.
B2	Represents a regionally oriented world of moderate population growth and local solutions to environmental and economic issues. The fertility rate is medium with low domestic migration and medium international migration.

**Table 2.** Area (km^2^) change for land cover types from 2000 to 2100 for the Northern Rio Grande River Basin by Integrated Climate and Land-Use Scenarios [24].

Land Cover	Scenarios				
	A1	A2	B1	B2	BC
Exurban	4022	1078	820	878	377
Suburban	5007	18,364	274	700	333
Urban	188	95	3	6	3
Natural Areas	−9217	−19,537	−1097	−1583	−712
Percent Natural Area Loss	−1.65%	−3.49%	−0.20%	−0.28%	−0.13%
